# Multiclass Convolution Neural Network for Classification of COVID-19 CT Images

**DOI:** 10.1155/2022/9167707

**Published:** 2022-04-28

**Authors:** Serena Low Woan Ching, Khin Wee Lai, Joon Huang Chuah, Khairunnisa Hasikin, Azira Khalil, Pengjiang Qian, Kaijian Xia, Yizhang Jiang, Yuanpeng Zhang, Samiappan Dhanalakshmi

**Affiliations:** ^1^Department of Electrical Engineering, Faculty of Engineering, Universiti Malaya, Kuala Lumpur 50603, Malaysia; ^2^Department of Biomedical Engineering, Faculty of Engineering, Universiti Malaya, Kuala Lumpur 50603, Malaysia; ^3^Faculty of Science and Technology, Universiti Sains Islam Malaysia, Nilai 71800, Negeri Sembilan, Malaysia; ^4^School of Artificial Intelligence and Computer Sciences, Jiangnan University, Wuxi 214122, China; ^5^The Affiliated Changshu Hospital of Soochow University (Changshu No. 1 People's Hospital), Changshu, Jiangsu 215500, China; ^6^Department of Medical Informatics of Medical (Nursing) School, Nantong University, Nantong 226001, China; ^7^Department of Electronics and Communication Engineering, SRM Institute of Science and Technology, Kattankulathur, Chennai 603203, India

## Abstract

In the late December of 2019, a novel coronavirus was discovered in Wuhan, China. In March 2020, WHO announced this epidemic had become a global pandemic and that the novel coronavirus may be mild to most people. However, some people may experience a severe illness that results in hospitalization or maybe death. COVID-19 classification remains challenging due to the ambiguity and similarity with other known respiratory diseases such as SARS, MERS, and other viral pneumonia. The typical symptoms of COVID-19 are fever, cough, chills, shortness of breath, loss of smell and taste, headache, sore throat, chest pains, confusion, and diarrhoea. This research paper suggests the concept of transfer learning using the deterministic algorithm in all binary classification models and evaluates the performance of various CNN architectures. The datasets of 746 CT images of COVID-19 and non-COVID-19 were divided for training, validation, and testing. Various augmentation techniques were applied to increase the number of datasets except for testing images. The images were then pretrained using CNN to obtain a binary class. ResNeXt101 and ResNet152 have the best F1 score of 0.978 and 0.938, whereas GoogleNet has an F1 score of 0.762. ResNeXt101 and ResNet152 have an accuracy of 97.81% and 93.80%. ResNeXt101, DenseNet201, and ResNet152 have 95.71%, 93.81%, and 90% sensitivity, whereas ResNeXt101, ResNet101, and ResNet152 have 100%, 99.58%, and 98.33 specificity, respectively.

## 1. Introduction

The emergence of various types of pneumonia, such as Severe Acute Respiratory Syndrome (SARS), had a substantial cause of fatalities worldwide and threatened the nation. Despite the latest technological development, it is difficult to predict and detect new emerging diseases [1]; pneumonia was estimated that 18% of 1.4 million children less than five years old die of pneumonia yearly [2]. Two billion people suffer from pneumonia annually. There were numerous outbreaks of novel infectious diseases that cause viral pneumonia. These diseases can infect animals, ranging from bird flu, swine flu, SARS, MERS, and Ebola. The number of COVID-19 outbreaks has increased four times the rate since the 1980s. The spike increase in epidemics has resulted in the World Health Organisation (WHO) repeatedly reminded that the time would come when an epidemic would strike us. On New Year's Eve 2019, China's health authorities had activated a profound response level after an outbreak of novel viral pneumonia in central China.

A rapidly growing number of people developed a dry cough and fever before being diagnosed with pneumonia. Some of the infected patients experienced severe illness, including death. The Chinese health authorities tried to trace its origin. The likely source was at a food market called Huanan Market, Wuhan, China [3]. The earlier reported 41 patients contacted with the disease stated that 27 had visited the food market. Chinese officials immediately shut down the market because it had happened before. In 2002, a coronavirus had emerged in a similar market in southern China, specifically in Foshan, Guangdong province, that spread to 29 countries and killed 800 people. After almost two decades later, the current coronavirus has infected 221 countries and territories with 106,295,553 cases and claimed 2,371,923 lives until 6 February 2021 [3, 4].

The SARS-CoV-2 is 120 nanometers (billionths of a meter) across. A hundred million viral particles are required to fit the head of a pin needle. However, human beings only need a few hundred particles to get infected and develop symptoms [5]. The indication is now widely known to us as COVID-19.

### 1.1. Background on COVID-19

Generally, viruses that make us sick originated in animals. Some viruses that cause influenza come from birds and pigs. HIV/AIDS comes from Chimpanzees. The deadly Ebola virus originated from bats. In COVID-19, the virus originated from bats, spread to pangolin, and finally infected humans. COVID-19 can transmit concealed and contaminate almost everyone, reproduce in its host, and simultaneously infect other hosts. The virus has been able to evade itself from our body's immune system. Like all viruses, they are mindless scraps of genetic materials, adapting to any environment through evolution. COVID-19 is the most significant public health crisis of the last hundred years. The RNA stores all the genetic information to reproduce. The composition of the COVID-19's RNA is more superficial than our human being's DNA. The short strand of the COVID-19 RNA is protected by a fatty outer membrane coat that breaks apart easily when it encounters soap and water [6].

Therefore, the nation is advised to do frequent and more prolonged handwashing activities. The outer membrane of the virus has protruding stud-like spikes like crowns. The spikes' function is to infiltrate our cells, which the vaccine creators are targeting. COVID-19 can be infected by breathing, coughing, and sneezing from one human to another. Once COVID-19 can obtain a new host, it invades the cells. The human cells have a fatal weakness where an enzyme called ACE2 controls the body's blood pressure on the surface. The COVID-19 virus spikes share a similar shape to attach themselves tightly to the ACE2 enzymes. Once the virus's binding onto the enzyme is complete, the human cells open, allowing the virus to slip into the cells and take control. The ACE2 enzymes are found throughout the body, including the throat, lungs, eyes, and nose. Therefore, hand wash as frequently as possible and resist the urge to touch the nose or rub the eyes. Once the virus penetrates the cell, it releases a genetic code and immediately take overs the cell. The virus then begins to generate endless copies of it and simultaneously infect more cells in the process. The virus can multiply itself a million-fold. Generally, any virus evades into the human body and starts reproducing; the human body's immune system can fend the evaders off and unleash an attack upon the virus. COVID-19 virus, however, can trick the human body's immune system and cover the spikes with a layer of glycan sugar and disguise itself as viral proteins, which help them evade the body's immune system. The COVID-19 virus devastates the human body with a swift, incognito, powerful attack; it is a battle between the COVID-19 virus and its immune system, which dictates its severity of illness. COVID-19 virus can silence the alarm of the human body's immune system, and thus most infected people start showing symptoms several days later. Hence, people exposed to a person infected with COVID-19 must quarantine themselves, regardless of whether the COVID-19 test is positive or negative. Some people have COVID-19 viruses in their bodies but never show symptoms. These people are known as “asymptomatic carriers”. They travel everywhere blissfully; unbeknownst that they are distributing potentially lethal viruses everywhere they go.

The difference between COVID-19 viruses and other coronaviruses is the ability of a person who does not show significant symptoms of the COVID-19 virus and infects others. SARS-CoV, a.k.a SARS: in 2002, an American businessman who traveled from China to Singapore transited in Vietnam was caught with symptoms similar to pneumonia during the flight and was immediately taken off the plane to a hospital in Hanoi. Subsequently, the businessman passed away; the medical staff treated him later, developed symptoms, and one of them died. SARS showed to be deadlier than COVID-19 as it killed 10% of the infected people. However, SARS displayed its symptoms first before it became infectious. The SARS virus cannot bind itself to the ACE2 enzymes in the human body's respiratory tract as efficiently as COVID-19 making it less infectious than the latter. Since the SARS virus could not hide its presence from the human body's immune system or bind itself efficiently, it did not cause a pandemic. There were 8,000 reported SARS cases and 774 deaths. In 2004, there were no new SARS cases reported.

Once the COVID-19 virus enters the body, it infects the nose's cells and the upper throat. Hence, the first symptom is usually a dry cough. Some viruses pass the throat and bind to the intestines' ACE2 enzymes, causing diarrhoea. When it travels through the windpipe and infiltrates the lungs, the virus is deadlier, subsequently binding itself with ACE2 enzymes, causing pneumonia-like symptoms, including fever, cough, chills, shortness of breath, and chest pain. The symptoms are indications that the body is battling against the COVID-19 viruses. The human body's innate immune system activates neutrophils, i.e., a type of white blood cells and macrophages. A healthy human being can rely on the innate immune system to keep the COVID-19 virus under control. However, some might experience severe illness because the COVID-19 virus has traveled from the throat to the lungs, causing the dry cough to be more severe; the person typically develops a fever over 100.4°F; shortness of breath develops bone ache. When the infection becomes severe, the T-killer cells are activated to destroy cells that COVID-19 viruses have infested. Plasma B cells release billions of antibodies and try to eliminate COVID-19 viruses. Since the COVID-19 virus is novel, the human body has never encountered the particular virus before. The antibodies require longer to develop into the exact shape that binds to the COVID-19 viruses' spikes. Therefore, once the antibodies are developed, they can be during the later stage of the illness. A healthy and active immune system is essential to overcome COVID-19 viruses. Hence, senior citizens over 60 years old tend to have weaker immune systems, and the adaptive response to the COVID-19 viruses is slow. Unexpectedly, most senior citizens' immune systems overreact while battling the COVID-19 viruses, creating a surge of aggressive immune cells that can damage the lungs and other organs in the body, causing them to be even weaker. Some COVID-19 patients recover, and some will not. COVID-19 patients who did not recover experience shortness of breath, and they get their lungs scanned by chest X-ray (CXR) or computed tomographer (CT).

The radiologists look for “ground-glass opacities.” The lungs' fuzzy spots are signs of the worst form of pneumonia called acute-respiratory-distress syndrome, or ARDS. Alveoli in the lungs fill with fluid, reducing the COVID-19 virus patient's ability to absorb oxygen, thus causing the patient to be breathless. The immune system surges the area with T-killer cells, antibodies, and cytokines eager to destroy the virus and simultaneously destroy healthy tissues. Once the alveoli in the lungs are filled with fluid, the body has insufficient oxygen to supply other organs, especially the brain. The patient is sent to the intensive care unit, where doctors and nurses are fully garbed with personal protective equipment (PPE). The patient gasps for air uncontrollably. The medical providers decide if the patient requires an oxygen mask, intubation, or use of ventilators depending on the severity of the illness. For patients who use ventilators, the possibility of dying is high. Patients who survived the ventilator could never be fully recovered. At this point, the immune system is not only targeting the lungs, but it has also spread all over the body through the bloodstream. The liver, kidneys, guts, and brain are simultaneously attacked. When the COVID-19 viruses travel through the bloodstream, they form blood clots and subsequently cause heart inflammation. Once the COVID-19 virus reaches the brain, it can cause seizures.

The pathology of COVID-1 also greatly resembles SARS with general symptoms of fever, dry cough, shortness of breath, and fatigue [1]. Chest X-ray images of distressed patients showed interstitial, alveolar opacities, and Ground Glass Opacity basically on the periphery of the lungs [1]. Most agencies worldwide adopted the Real-Time Polymerase Chain Reaction (RT-PCR) to detect the virus consistently. The problem that cannot be overlooked is the RT-PCR, which is dependable enough to eliminate the false positives and false-negative results. The number of false negatives may cause an exponential increase, defeating the purpose of fighting against the pandemic.

The radiologist has utilized chest CT to detect COVID-19 infection by using specific and consistent features like ground glass opacities at the early stage and pulmonary embolism with linear consolidation at the later stage. Since COVID-19 has similar symptoms to pneumonia, detecting both of these viral lung diseases is confusing. Research depicted that Chest CT has a higher sensitivity for the diagnosis of COVID-19 compared to RT-PCR tests [7]. During the pandemic, the method of evaluating CT images of possible COVID-19 patients is manual and labor intensive. Therefore, an alternative solution is needed to automate the process and obtain precise and early detection to control the spread of COVID-19.

Convolutional Neural Networks have been applied for object detection, identification, segmentation, and classifications even for various diseases and contributed to the healthcare industry. Top-performing models like ResNet50 [8], Inception [9], ResNeXt [10], and DenseNet [11] can be applied to classify images. The weights of these models are trained using millions of images for similar recognition works. The models can be improved by using Batch Normalisation, regularisation, and fine-tuning the hyperparameters of the CNN. Based on the study conducted by Narendra et al. [12], the proposed model was based on VGG16 and ResNet50 with a 5-fold cross-validation for binary and multiclass classification produced an average accuracy of 88.52%. The research showed that the proposed model worked very well on binary classification. However, performance declined when they included pneumonia as the third class in the study [12]. A research study conducted by Sergio and Patricia [13] described a hybrid approach on a modular artificial neural network integrating with fuzzy logic to diagnose pulmonary diseases such as pneumonia and lung nodules with a maximum of 99.83% accuracy for lung diseases classification.

Furthermore, the RT-PCR relying upon gene primers' application may hinder detecting true positive cases if the COVID-19 genome mutates. Therefore, the limitation of RT-PCR results needs to be interpreted with caution. Besides that, RT-PCR requires two days to achieve results that may or may not be accurate. Therefore, such an adjournment affects the start of isolation and treatment for the COVID-19 patient.

This research applied the common loss versus epoch and accuracy versus epoch line graph; besides that, GradCAM was used to visualize the activation of the deep learning models as a heatmap overlaid on the COVID-19 CT images. It was completed at various checkpoints during the training to ensure optimum hyperparameters. The study depicted that the deep learning models were initially pretrained using the ImageNet database and implemented the models' pretrained weights as the initial state to kickstart the training, specifically for the COVID-19 image set.

## 2. Convolutional Neural Networks and Deep Learning Related Works

The rapid progress of medicine combined with various computational approaches has led to numerous developments. One of the branches of computer science is artificial intelligence (AI). Medical imaging focused primarily on radiology, cardiology, and pathology. A radiology example includes automated microcalcifications and masses on mammography lung nodules on chest X-rays and CT scans [14]. Computer-Aided Diagnosis (CAD) became a backup diagnosis for specialists and physicians. CAD applications are myriad and provide hypotheses based on the algorithm to identify abnormalities within the organs such as breasts, lungs, heart, gastrointestinal tract, neuro, and whole-body imaging. However, if the algorithm in the CAD system lacks accuracy, it would create false positives and misdiagnose or misidentify the patient for having an abnormality, which ultimately ends up otherwise. Artificial Intelligence (AI) and the development of Convolutional Neural Network (CNN) created the ability to identify images and detect objects on the image. The human body images were created for medical procedures and diagnosis in medical imaging.

The development of CAD systems is time-consuming and labor-intensive. Therefore human beings are leveraging Artificial Intelligence (AI) tools to improve CAD system efficacy. The CAD algorithm is divided into two parts, i.e., initial lesions identification and false-positive reduction. The initial lesion identification consists of preprocessing, segmentation of body regions, candidate generation, and feature extraction. In contrast, the false-positive reduction consists of the radiologist's visual presentation of CAD findings. The interpretation of CAD systems has been challenging. Once the medical data and reference standard have been obtained, the data must be annotated [14]. The abnormality and the precise location must be determined by a professional. The images are best annotated by several experts so that the assessment's probability is to identify the lesion's location is accurate. Hence, the best evidence usually comes from large datasets, but they are usually unattainable unless a well-funded study.

Deep learning was introduced to improve neural network types by having more layers to permit high levels of abstraction. Deep learning successfully recognized objects in authentic world images and learned the features from the training data. Hence, some researchers found that CAD systems that require hand-chosen parameters and hand-crafted features to be manually selected and annotated are costly, time-consuming, fragile, and unreliable when applied to new data [14]. Deep learning can avoid such manual hand-tuning procedures.

Chest X-Rays (CXR) and Computer Tomography (CT) images have proven to be valuable resources to diagnose COVID-19. Ioannis D. Apostolopoulos and Tzani Bessiana [15] implemented feature extraction via transfer learning to extract features and include them into the new network that performs classification tasks like VGG19, MobilenetV2, Inception, Xception, and ResNet v2. Their research showed VGG19 achieving the highest accuracy in the binary classification of 98.75% and the multiclass classification of 93.48%. Ozturk T. et al. [16], based on the DarkNet architecture, established the DarkCovidNet, which produced a classification accuracy of 98.8% for binary classification and 87.02% for multiclass classification. Ohata et al. [17] utilized two datasets with feature extraction and transfer-learning by selecting different CNN architectures like VGG, Inception, ResNet, NASNet, Xception, MobileNet, and DenseNet that produced excellent results on the ImageNet dataset. Then, they selected different configurations from the trained CNN architectures, removed the fully connected layers from these configurations, and maintained convolutional and pooling layers. This is necessary to convert the CNN architectures to feature extractors for CXR images using the subdatasets as an input. The final classification applied machine learning algorithms such as Support Vector Machine (SVM), K-Nearest Neighbour (KNN), Bayes, Random Forest (RF), and Multilayer Perceptron (MLP). The author highlighted the different types of classifiers: KNN is instance-based, RF is a decision tree method, MLP is based on neural networks and others. The MobileNet-SVM architecture produced the highest accuracy of 98.5% for the first dataset, and DenseNet201-MLP achieved the highest accuracy of 95.6% for the second dataset.

The research aims to develop a Deep Learning-based classification model to automate the binary classification of COVID-19 and Non-COVID-19 lung CT images. The first stage is image classifications using a feasibility study on COVID-19 and DL algorithms. The second stage is to develop classification models by preprocessing the datasets to ensure the data are standardised. Data augmentation was carried out to increase the number of datasets, and a transfer learning strategy was implanted to train the datasets. The third stage is to evaluate the performance of the trained models.

The method is based on the supervised Deep Learning algorithms to conduct a binary classification of CT images of COVID-19 vs. non-COVID-19. The methodology was categorized into three stages, i.e., (i) research methods, (ii) data collection and acquisition, and (iii) data analysis.

### 2.1. Tools and Materials

In this study, an 9th Gen Intel® Core™i5-9300H (4C/8T, 2.4/4.1 GHz, 8 MB) with Windows 10 platform was applied to conduct the study. The algorithm of the study used Python on Microsoft Visuals Studio Code with PyTorch framework. In addition, Google Colab Virtual Machine was implanted for image training, validation, and testing.  Table 1 describes the summary of Google Colab GPU. [18].

### 2.2. Methods and Model Classification

The proposed research applies the binary classification method to distinguish between the COVID-19 and non-COVID-19. The original datasets are divided into an 8 : 1 : 1 ratio for training, validation, and testing. The input images are translated, scaled randomly into different sizes, rotated and flipped horizontally and vertically by data augmentation. The augmented images underwent transfer learning using the classification model. Finally, the images were localized using GradCam or Heatmap.

### 2.3. Data Collection

The COVID-19 CT images were collected from medRxiv and bioRxiv published from 19 January 2020 to 25 March 2020. The data were made available on GitHub, an open-source repository that hosts source codes of software development projects in different programming languages. Each CT image was associated with captions to differentiate between COVID-19 CT and non-COVID-19 CT, as described in Table 2. [19]. The total number of COVID-19 CT images is 746, 349 of them are COVID-19 images, and 397 are non-COVID-19 images. The images were extracted using PyMuPDF from research papers [19].

### 2.4. Data Preprocessing & Augmentation

The data were collected from various studies: the images' dimensions, contrast, and intensity needed to be consistent. Some images were unsuitable for preprocessing as the lung density appeared utterly black. However, the study aims to produce a robust model for noise artefacts and overcome input variations. The images obtained had different dimensions and were resized to 224 × 224 pixels. During image resize, to avoid compromising the details of the images, the ratio aspects of the images remained. Firstly, the sizes of the input images were adjusted to the preset size. Then a blank square with 224 pixels was attached to create a new image. The resized image was attached to a square using Python's “Pillow (PIL)” image processing package. Finally, the images were normalized using minimum-maximum normalization techniques with mean and standard deviation. The normalization method eliminates the bias from the features and datasets within the range.

The study consists of a transfer learning strategy with deep learning CNN models, which requires a significant number of data to prevent overfitting in a complex network. Data augmentation can increase the quantity of the training samples using rotations, horizontal flips, vertical flips, translation, color jitters, random perspective, random affine, and auto augmentation in the *x* and *y*-axis [20]. The color jitter arbitrarily alters the brightness, saturation, hues, contrasts, and other properties of the images. The random perspective transform function allows the image's perspective to be randomly distorted and scaled at different angles. The rotation transform function rotates the images within the angle of 30°–70°. These transformations maintain the quality of the image and would not hinder the radiologist's ability to interpret.  Table 3 describes the number of datasets after data peprocessing and augmentation [19].

### 2.5. Training

After preprocessing and augmentation of the image, transfer learning techniques were applied. The CNN networks above were trained on the ImageNet dataset. The pretrained models functioned as feature extractors to avoid learning from scratch. The network was initialized, and the new final convolutional layer (FCL) was fine-tuned. The FCL was modified to generate a task-specific model. PyTorch frameworks such as ResNets, DenseNets, GoogLeNet, and ResNeXt were selected according to the error rate on ILSVRC and models' architecture. The deterministic algorithm currently supports all PyTorch models except AlexNet and VGGNet because they are the older models in CNN architectures. Hence, AlexNet and VGGNet were not included in the study.

GoogleNet is known as Inception-v1, was the CNN architecture network that won the ILSVRC′14 competition, followed by VGGNet as the First Runner up. It has 22 layers and looks into computation efficiency using the inception module and stacked the modules on top of one another. It does not have fully connected layers (FC) and thus reduces the number of parameters. The number of parameters in GoogleNet is 5 million, which is 12 times lesser than AlexNet and 27.6 times lesser than VGGNet. GoogleNet top 5 error rate is 6.7%.

**Figure 1:**
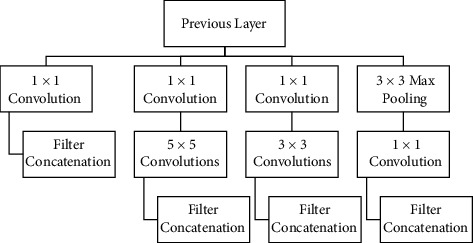
The architecture of GoogleNet by Szegedy et al. [9].

The inception module designed a local network topology, which created a network and stacked the local topologies on top of each other. GoogleNet applied parallel filter operations on the input of the previous layer, which consisted of various receptive field sizes of convolution, e.g., 1 x 1CONV layer, 3 × 3 CONV layer, 5 × 5 CONV layer, and 3 × 3 pooling operation as shown in Figure 1. These different filter layers provided different outputs, and all filter outputs were concatenated together depth-wise. Finally, it created one tensor output to be continued in the next layer. The problem with this process is computational complexity. A naïve inception module can maintain the spatial dimensions but increase the depth within the network. The entire CONV operations are 854 million. The complexity resulted in the operation being expensive to compute. The pooling layer is an additional problem for every module. The pooling layer preserved feature depth; thus, the total depth after the concatenation increased at every layer. GoogleNet addressed these problems by implementing “bottleneck” layers that reduce feature depth by using a 1 × 1 CONV layer. It projected the feature maps to a lower dimension, preserving the spatial dimensions and reducing the depth. The “bottleneck” layers alleviated the expensive computing and reduced operations to 358 million compared to the 854 million for the naïve version. Figure 1 describes the feature maps of GoogleNet [9].

**Figure 2:**
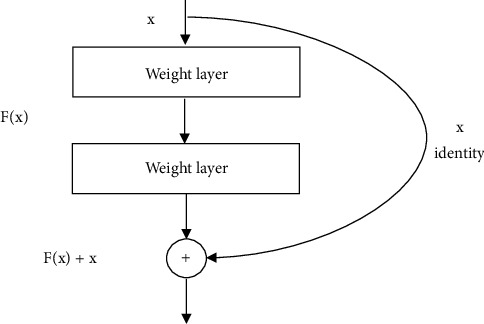
Residual learning building blocks adapted from [8].

Residual Network, also known as ResNet, is a more profound CNN architecture with a 152 layer model in ILSVRC′15 with a top 5 error rate of 3.57%. ResNet is the architecture that won in both ILSVRC′15 and COCO competitions. However, a deeper network does not necessarily mean better CNN architecture. The problem is not overfitting but an optimization issue. Deeper models are harder to optimise. The solution was to copy and learn the layers from the shallower model and set additional layers to identify and map. The hypothesis from the author of ResNet applied network layers to fit a residual mapping instead of directly trying to learn and fit a desired underlying mapping. A complete ResNet architecture has stacked residual blocks, and every residual block has two 3 × 3 CONV layers. Then, double the number of filters periodically and reduce the spatially by half. The convolutional layer was added initially, and fully convolutional (FC) networks were not included. The global average pooling layer will average everything spatially and be input into the last 1000 way classifier. ResNet34, ResNet50, ResNet101, and ResNet152 are the numeric values that indicate the total depth of the architecture neural network. ResNet above 50 layers used the “bottleneck” layer to improve efficiency, similar to GoogleNet. ResNet used batch normalization after every CONV layer, implemented Xavier/2 initialization, SGD and momentum are 0.9, the learning rate is 0.1 divided by 10 when the validation error plateaus, minibatch size of 256 without dropout.  Figure 2 describes the building blocks of ResNet adapted from (He et al., 2016) [8].

**Figure 3:**
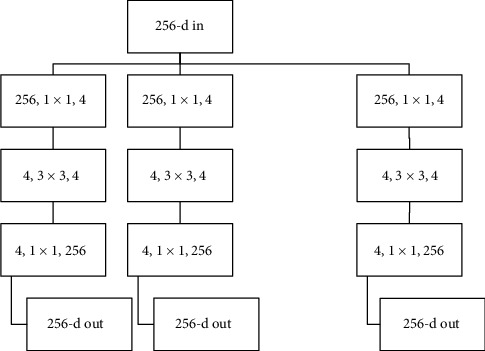
A block of ResNeXt adapted by [10].

The same creator of ResNet created ResNeXt and won the 1st Runner Up of the ILSVRC′16 classification task. ResNeXt is the next dimension on top of ResNet, also known as the “cardinality” dimension. It increases the width of the residual block through multiple parallel pathways similar to the GoogleNet/Inception module by implementing three simple steps of splitting, transforming, and aggregating. The neuron inside the neuron controls complex feature numbers. ResNeXt obtained the top 5 error rate of 3.03%, displaying a 0.54% gain compared to its predecessor.  Figure 3 shows a block of ResNeXt adapted by Xie et al. [10].

**Figure 4:**
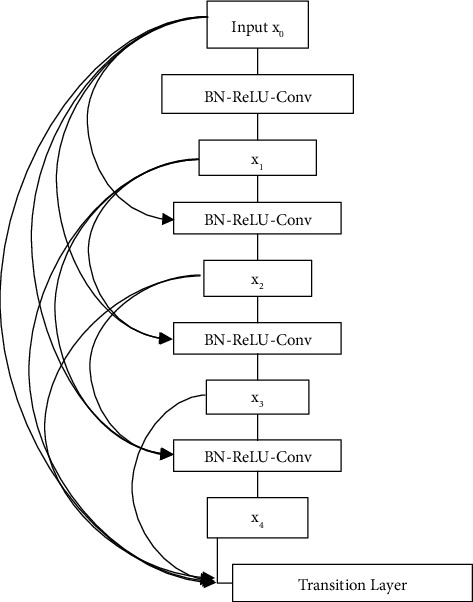
DenseNet 5-layer dense block with each layer takes all feature maps as input by Huang et al. [11].

**Figure 5:**
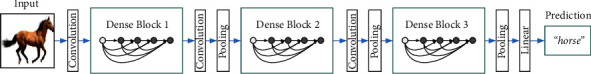
A DenseNet with three dense blocks by Huang et al. [11].

DenseNet is also known as Densely Connected Convolutional Network. It means having dense blocks where each layer is connected to every other layer in a forward feed manner, alleviates vanishing gradient, strengthens feature propagation, and encourages feature reuse. The dense block has multiple connections, and each feature map learned is input to multiple layers and used multiple times.  Figure 4 is the five-layer feature map of DenseNet, and Figure 5 shows the 3 five-layer dense blocks combined to create a complete DenseNet [11].

### 2.6. Deterministic Implementation

The deterministic algorithm in deep learning is used to ensure the results are replicable. The algorithm's primary purpose is to achieve replicability. The deterministic implementation runs under fixed experimental conditions and ensures that the output is identical to any input [21]. It is one of the most studied and practical algorithms because it can run on machines efficiently. The study implemented deterministic implementation on ResNets, GoogleNet, DenseNet, and ResNeXt. The sources of nondeterminism were typically from GPU. Many operations performed on the GPU are nondeterministic by default [21]. The transition in the deep learning environment can be random. The deep learning models employ stochastic policy during training, and before training, the neural network weights were arbitrarily decided.

### 2.7. Hyperparameters

Once the neural networks were defined, the hyperparameters were trained to achieve accuracy. The learning rate was set at 0.01 required a prolonged training period. The momentum was set at 0.9 to prevent oscillations. The parameters allowed the network to adapt and identify the next step from the previous training, which also controlled the speed during training. Batch size is the number of subsamples fitted to the network in every epoch. The number of epochs was 300 since the number of datasets is small. The training will stop if the validation loss values depict no progress within ten epochs. The setting of the hyperparameters was essential. Thus, a few trials were done to ensure the most suitable number of the epoch. Initially, the models were all set to run at 100 epochs, but the gap between the training and validation losses was depicted that the model is underfitting, and the model is still able to learn. Hence, 300 epochs were executed when the training and validation losses were similar.

The SGD optimizer minimizes the loss function while updating the computed gradient parameter by training the network. Backpropagation calculated the gradients during training, and the optimizer learned with the available gradients [19]. The weight decay was specified at 0.1, decaying the learning rate by a factor of 0.1 every nine epoch. Cross-entropy loss increased the predicted probability diverged from the actual label to reduce the loss to improve the training.

### 2.8. Performance Evaluation

During classification, the algorithm for prediction is evaluated to determine the efficiency of the classification model. This is essential to determine whether the trained model and the classifier can execute arbitrary test data. The general practice is to apply predictive accuracy as the evaluation benchmark. However, accuracy is insufficient to determine the reliability of the classification model.

The confusion matrix is a table created to describe the performance of the classification model, also known as the classifier [22]. The table can be divided into four categories for binary classifiers, i.e., True Positives, False Positives, True Negatives, and False Negatives, arranged in a 2 × 2 table. True Positives show that the model was predicted and matched with the actual label as “Non-COVID-19”. True Negatives are predicted and matched with the actual label as “COVID-19 positive”. With the confusion matrix table, the following evaluation indicators can be obtained: accuracy, misclassification rate/error rate, true-positive rate/sensitivity/recall, false-positive rate, true-negative rate/specificity, precision, prevalence, and F Score. Since accuracy alone could not determine the classifier's performance, the evaluation metrics were computed in detail.

### 2.9. Localisation of Abnormalities

Gradient-weighted Class Activation Mapping, also known as Grad-CAM, is a “visual explanation” for decisions from a large class of CNN-based models. This technique implemented the targeted concept's gradients and highlighted regions in the image for prediction to create a coarse localization map [23]. Therefore, this technique was applied to highlight the classifiers' regions identified and analyzed if the classification models were feasible.

## 3. Results and Discussions

### 3.1. Training and Validation

The dataset was trained with various pretrained architectures: ResNets, GoogleNet, ResNeXt, and DenseNet. ResNet was performed thrice with different depth layers such as ResNet50, ResNet 101, and ResNet 152. The remaining GoogleNet, ResNeXt, and DenseNet were performed once. This section recorded all the results from the various CNN architectures.

Each epoch has 3,588 images with a batch size of 64. It consists of the training loop and the validation loop. After each training, the validation loop was evaluated, and the cycle was repeated until the maximum epoch was achieved. Once the training and validation were completed, the models were tested for sensitivity, specificity, precision (PPV), negative predictive value (NPV), accuracy, precision, and F1 score.

The losses of the training and validation of the transfer learning models were shown below:

The accuracy of the training and validation of the transfer learning models were depicted below:

The process of training, validation, and testing the neural network is to predict arbitrary datasets provided to the classification model correctly for future applications. The goal is to achieve low training and validation loss. If the training loss is low and the validation loss is high, it is overfitting. If both the training and the validation loss are high, it is underfitting. There could be two possibilities if the training loss is higher than the validation loss. The first one would be that the validation dataset is leaked into the training dataset. The model needs to be regularised for overfitting circumstances, or a more straightforward model should be chosen to train and validate; for underfitting, a more robust model is required to train and validate.

An example would be that a particular *x* and *y* present in the validation datasets are also found in the training dataset. Another example would be some form of the data from the validation dataset being leaked into the training dataset, like the normalized input consisting of R, G, and B, which uses the standard deviation and the mean of the training dataset only. The second example would be that the training dataset is more complex than the validation dataset. When data augmentation was applied, the images were distorted, rotated, scaled, and modified to create obstacles for the algorithm to learn. The third example is that some connections might be disabled during training to limit the algorithm's capabilities; resume full capabilities and connections to run the validation and the test sets. Hence, the possibility of the validation loss would be lower than the training loss.

The loss or the cost measures the model error. The purpose is to lower the losses to improve the models' performances. The training loss depicts how well the model trains the data, while the validation loss depicts how well the model fits new data. The graphs are performance learning curves calculated on the metric the model was evaluated and selected. High bias happens when the algorithm learns it could not accept all the related information, failing to grasp the model's complexity. Underfitting occurred when the algorithm could not model the training and new data, which resulted in high error values. When the model is too complex and does not represent the simple patterns in the data, the variance is high. The algorithm manages to train the data but poorly generalizes the new data.

**Figure 6:**
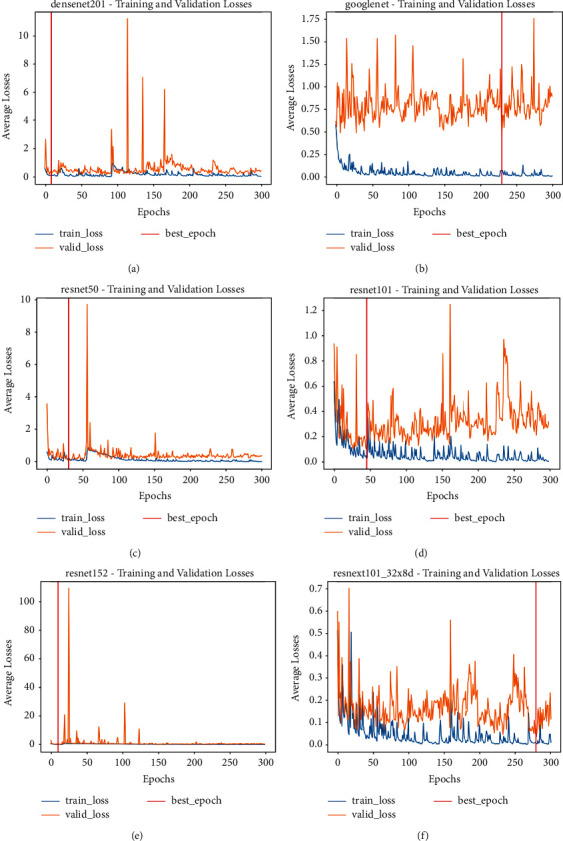
The training and validation losses during transfer learning. The blue line represented training losses, and the orange line represented the validation losses. (a) DenseNet201. (b) GoogleNet. (c) ResNet50. (d) ResNet101. (e) ResNet152. (f) ResNeXt101.

**Figure 7:**
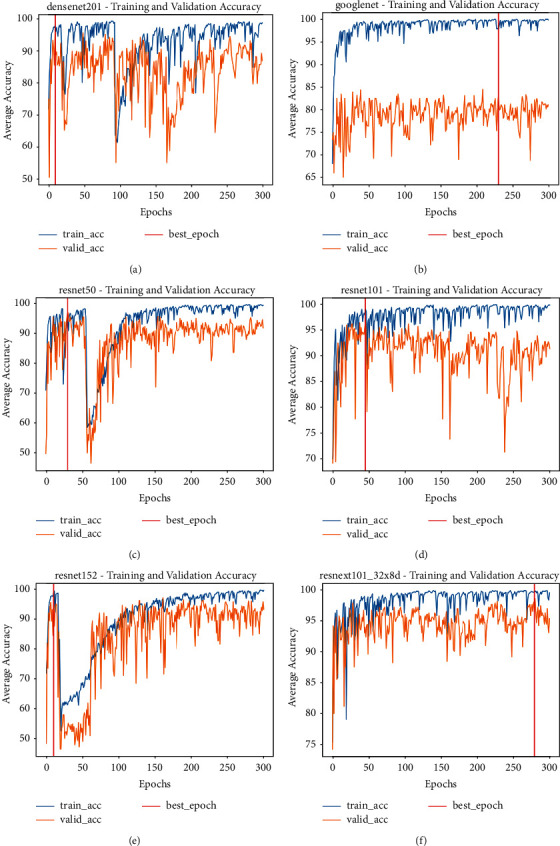
The training and validation accuracy during transfer learning. The blue line represented training accuracy, and the orange line represented validation accuracy. (a) DenseNet. (b) GoogleNet. (c) ResNet50. (d) ResNet10. (e) ResNet152. (f) ResNeXt101.

The weights and biases of the neural network were determined through training. Figures 6 and 7 each depicted the losses and accuracies for training and validation. The classifiers in Figure 6 were trained for 300 epochs, and at any point of the training and validation that ten epochs consecutively did not improve, the algorithm stopped early. From the graphs in Figure 6, the blue line represents training loss, and the orange line represents validation loss. It showed that the validation loss fluctuates. These were obvious in the cost function shown in the DenseNet, ResNet 50, and ResNet 152. It showed that the loss was high and fluctuated, which means it did not decrease.

The neural network model can capture the training data very well, but the lack of performance in the validation data showed overfit. Over time, the training loss was reduced for GoogleNet, ResNet 101, and ResNeXt, achieving low error values. However, the validation losses were observed to reduce until a specific turning point increased again. The neural network models showed that the train and validation curves have gaps between them, which mean they function like the datasets are from various distributions. The validation loss fluctuates noisily around the training loss, depicting that the neural network models struggle to model these validation datasets.

### 3.2. Training Parameters and Training Time

**Figure 8:**
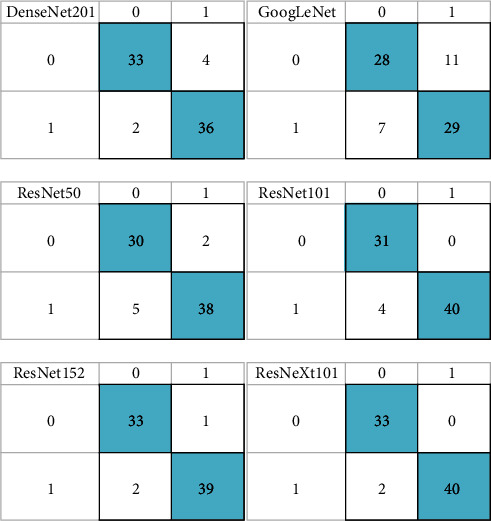
Confusion matrix for DenseNet201, GoogLeNet, ResNet50, ResNet 101, ResNet152, and ResNeXt101.

Table 4 and Figure 8 describe the training parameters and the training time per epoch of all the trained neural networks. In every epoch, the total number of images loaded into the networks was 3,588 images with a batch size of 64.

Once the training loops were completed, the validation loops continued; hence, the repetitive cycle continued at each epoch to prevent overfitting. DenseNet, GoogleNet, ResNet50, ResNet101, ResNet152, and ResNeXt101 validation and training losses are displayed in Figure 6, and the validation and training accuracy in Figure 7, respectively.

### 3.3. Confusion Matrix

After completing the training and validation set, the confusion matrix was applied to each trained neural network plotted Class 0 as “Non-COVID-19” with 40 images and Class 1 as “COVID-19.” with 35 images. The test images were divided into the following prediction results: TP, TN, FP, and FN [24]. Figure 8 describes the confusion matrix for DenseNet201, GoogLeNet, ResNet50, ResNet 101, ResNet152, and ResNeXt101.

ResNeXt 101 had two wrong classifications, followed by ResNet 152 with three incorrect types, and ResNet 101 with four misclassifications. GoogLeNet had the most with eleven misclassifications.

### 3.4. Performance Metrics

After training and validation, the models were tested using an unseen dataset. The accuracy, sensitivity, specificity, PPV, NPV, and F1 scores were recorded in Table 5.

Based on Table 5, ResNeXt101 showed a sensitivity of 95.17% with two misclassifications of COVID-19 images, whereas DenseNet201 had achieved a sensitivity of 93.809%, and ResNet152 attained a sensitivity of 90%, both with two wrong COVID-19 images classified. ResNet 101 attained a sensitivity of 88.57% with four false classifications, and ResNet 50 gained 86.19% with five incorrect categories of COVID-19 images. Lastly, GoogleNet had a sensitivity of 80.95% with seven wrong COVID-19 image classifications.

PPV, also known as precision, indicated how often the model predicted COVID-19 was correct. PPV considered the models to be performing well. Suppose the precision value was not good despite the very high models' accuracy. The models would not be considered good. ResNeXt101 had achieved 100% PPV, whereas ResNet101 had achieved 99.47% follow up by ResNet152, which achieved 97.93%. GoogLeNet achieved the lowest precision of 72.03%, with seven wrong classifications of Non-COVID-19.

### 3.5. GradCAM Visualisation

The GradCAM Visualisation was implemented to understand the network's region [23]. Based on the performance metrics, the model with the highest accuracy is ResNeXt101, with 98% accuracy.

Table 6 shows the GradCAM visualization. The red region represented the highest region of interest [25]. DenseNet201, GoogLeNet, ResNet101, and ResNeXt101 detected the area of interest in the middle of images as COVID-19 images. DenseNet 201 and GoogleNet have the highest percentage with 99.80% and 99.99% for detecting the image as positive for COVID-19. ResNeXt101 and ResNet 101 detected the image as COVID-19 positive with 98.04% and 94.48%. ResNet152 detected the image as a COVID-19 image with 95.31%. Still, it caught the area of interest skewed more towards the right of the pictures, different from the rest of the models. However, ResNet50 detected the image with 83.16% as Non-COVID-19, with the image appearing to have three red spots of the region of interest. The model is still inadequate in detecting the region of interest to be COVID-19 positive images.

### 3.6. Research Limitations

The research applied small datasets to train the CNN architectures, a total of 746. Due to the model complexity: either overfitting or underfitting. The quantity of the dataset was further reduced when they were divided into three categories to train, validate, and test the neural networks. The problem of data scarcity is often responsible for poor performance, which outcomes in incomplete projects.

Six different data augmentation methods were performed on the training and validation datasets to compensate for the lack of quantity of the dataset to train the neural network to be more robust and versatile in classifying COVID-19 and Non-COVID-19 images. However, based on the training and validation curves, the validation loss described that the neural network models picked up noises that cause overfitting. Overfitting in the validation loss indicates that more training examples are required to improve the model performance on the unseen data. The batch normalization technique had been utilized, but it only produced a slight regularisation effect. Therefore, the fluctuation of peaks occurred in Figure 6. Dropout can be used to increase the regularisation effect. The research also conducted cropping and rotation of the images as data augmentation operations to create more data for the algorithm to learn. The algorithm overcropped the images, combining with rotation exacerbated the loss of essential clues in the images. Hence, the algorithm created arbitrary images that excluded important information required to learn and classified it as COVID-19 or non-COVID-19 images. Cross-validation can be applied to increase the amount of training data. The learning rate of the algorithm can be lowered to improve the performance of the graph in Figure 6. The CNN optimizers can be improved instead of stochastic gradient descent (SGD) with momentum; ADAM can improve the algorithm.

The deep learning models require significant hardware and computational costs to train models efficiently. Google Collaboration is a free online GPU that could not cover the neural networks due to limitations.

## 4. Conclusion and Future Works

The pandemic of COVID-19 has adverse effects all around the world. It especially challenged the healthcare system due to its highly infectious nature. The vaccine has been distributed worldwide, and many people have received it globally. Although the people are vaccinated, reducing the transmission rate of COVID-19 by isolating suspicious individuals from healthy ones continues to interrupt the transmission. However, the virus mutates, and new variants emerge and disappear. The variants of concern are Alpha, Beta, Gamma, and Delta variants. The Delta variant is becoming the dominant variant, and vaccines cannot guarantee its effectiveness towards the delta variant and other COVID-19 mutations. The viral tests like the nucleic acid amplification (NAATs) and the antigen tests are used to recognize current infection but cannot identify which variant an infected patient has [26]. CT is widely applied in the emergency department and patients who perform extra thoracic CT.

This binary classification study between COVID-19 CT images from Non-COVID-19 CT images applies various transfer learning models and comprehensive analysis to distinguish the images by machines automatically. Firstly, the images were divided into training, validation, and testing. The images divided into training and validation categories were augmented to increase the datasets. The images under the testing category were not augmented. Then the binary classification of the augmented dataset was implemented on ten different pretrained models. The results have shown that ResNeXt101 had achieved the highest accuracy of 98%. ResNet101, ResNet152 had an accuracy of 94.44%. DenseNet201 achieved 91.78%. GoogLeNet had the least number of training parameters with 5,601,954, and the training time per epoch was the shortest, with 31.31 seconds per epoch. ResNeXt101 superseded the expectation and performed better than the other neural network models. Although the architecture achieved the best level of accuracy, it has the highest computational cost.

The confusion matrix generally depicts the performance of the classification models on the testing data. The performance metrics were computed to quantify overall and individual class performance. All classification models performed better in classifying the Non-COVID-19 images than the COVID-19 images. The following stage was to apply GradCAM to reveal which models were emphasized. The blue and red on the images are high-intensity regions which are helpful to understanding the decision made by the classification models and further fine-tuning for future improvements. ResNeXt101, ResNet101, ResNet152, and ResNet50 are models with higher PPV and lower NPV percentages, except DenseNet and GoogLeNet. Neural network models with higher PPV and lower NPV better classify COVID-19 images than Non-COVID-19 images.

GradCAM was implemented to visualize the neural networks and reveal the area of interest. The localization approach was helpful for the improvement of the trained models.

This research currently utilizes the standard optimizers in the algorithm: gradient descent and stochastic gradient descent; other CNN optimizers such as ADAM, SWATS, Adabound, LookAhead, and Adabelief may be used to train the model to improve the results in future works. The adaptive moment estimation (ADAM) could be created to prevent the learning rates from missing the minimum by decreasing the velocity, rectifying vanishing learning rates. On the other hand, SWAT was suggested due to the low ADAM performance as compared to Stochastic Gradient Descent (SGD). SWAT is a hybrid optimizer method that applies an adaptive method followed by a switch to the SGD during the appropriate time of the algorithm to produce optimum results. Adabound is an ADAM variant designed to be more robust to an extreme learning rate. The idea of this optimizer is to implement adaptive optimization and employs dynamic bounds on learning rates to achieve a gradual and smooth transition from adaptive methods to SGD. LookAhead is an optimization algorithm that chooses a search direction by looking ahead of the sequence of fast weights generated by another optimizer, whereas Adabelief was suggested to achieve fast convergence as an adaptive method, good generalization as an SGD, and training stability. The intuition of the optimizer is to implement the step size according to the belief in the current gradient direction. All these optimizers mentioned above could be utilized to further improve the results. Currently, the data augmentation generated 6 times more images than the original for training. The data augmentation may cause the model to overemphasize the same image features. Thus, less aggressive data augmentation should be able to counter this effect. Some of the augmented images are overcropped, and a few of the cropped images are rotated, exacerbating the loss of essential features in the images. To counteract this, the key strategy is to include more comprehensive training datasets [27–35].

## Figures and Tables

**Table 1:** Google colab CPU and GPU specifications.

No	Parameter	Google colab
1	GPU	Tesla T4
2	GPU memory	16 GB
3	GPU memory clock	1.59 GHz
4	Performance	8.1 TFLOPS
5	Support mixed precision	Yes
6	GPU release year	2018
7	No CPU cores	2
8	Available RAM	26.75 GB
9	Disk space	358 GB
10	CPU model name	Intel(R) Xeon(R)
11	CPU freq.	2.30 GHz
12	CPU family	Haswell

**Table 2:** The datasets were divided into training, validation, and testing with an 8 : 1 : 1 ratio, respectively.

Classes	Datasets	Training set	Validation set	Testing set
COVID-19	349	280	35	34
Non-COVID-19	397	318	40	39

**Table 3:** The number of datasets after data preprocessing and augmentation.

Classes	Total datasets	Training sets	Validation sets	Testing sets
COVID-19	2094	1680	210	204
Non-COVID-19	2382	1908	240	234
Total	4476	3588	450	438

**Table 4:** Total training parameters for all neural network models with computed time per epoch at batch size 64.

CNN architecture	One epoch (seconds)	Number of parameters
DenseNet201	1429.9926	18,096,770
GoogLeNet	31.3082	5,601,954
ResNet 50	360.9115	23,512,130
ResNet 101	306.4787	42,504,258
ResNet 152	1663.329	58,147,906
ResNeXt101	32.7611	86,746,434

**Table 5:** The performance metrics on the test sets.

CNN architecture	TPR sensitivity	TNR specificity	Positive predictive value (PPV) precision	Negative predictive value (NPV)	Accuracy	F1 score
DenseNet201	93.809	90.00	89.14	94.323	91.78	91.41531
GoogLeNet	80.952	72.50	72.034	81.308	76.44	76.23318
ResNet 50	86.19	95.83	94.764	88.803	91.33	90.27431
ResNet 101	88.571	99.58	99.465	90.875	94.44	93.70277
ResNet 152	90	98.33	97.927	91.829	94.44	93.79653
ResNeXt101	95.714	100.00	100	96.386	98.00	97.81022

**Table 6:** GradCAM visualisations.

Models	Classes scores [Class0, Class1]	Original image	After GradCAM
ResNeXt101	98.0389% COVID-19, 1.9611% non-COVID-19	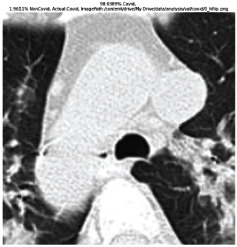	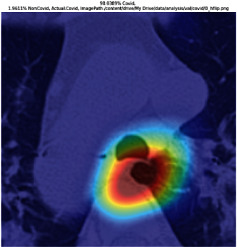
ResNet152	95.3125% COVID-19, 1.6875% non-COVID-19	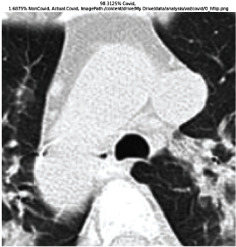	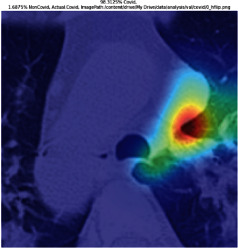
ResNet101	94.4808% COVID-19, 5.5192% non-COVID-19	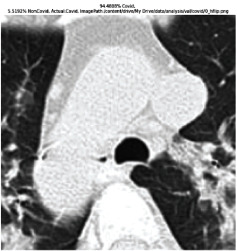	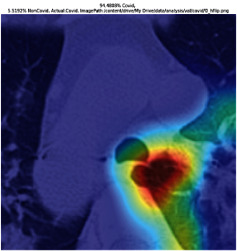
ResNet50	16.8379% COVID-19, 83.1621% non-COVID-19	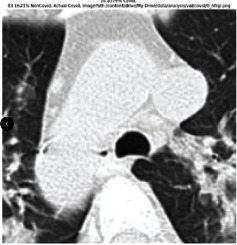	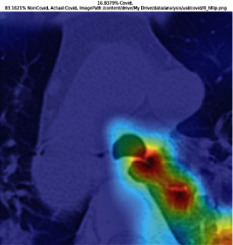
DenseNet201	99.7980% COVID-19, 0.2020% non-COVID-19	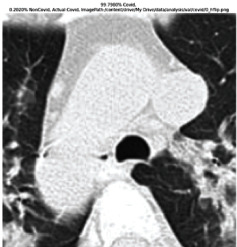	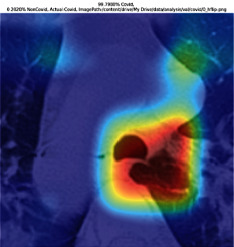
GoogLeNet	99.9991% COVID-19, 0.0009% non COVID-19	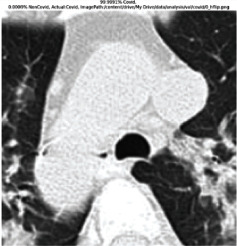	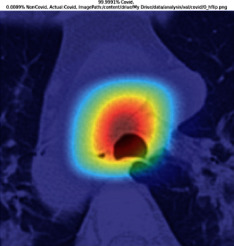

## Data Availability

All the data are available in the list of references.
